# Persistent diarrhea following catheter ablation for atrial fibrillation: A lesser-known complication of left atrial ablation procedures

**DOI:** 10.1016/j.hrcr.2021.06.009

**Published:** 2021-07-02

**Authors:** Teiichi Yamane, Osamu Inaba, Eri Hachisuka, Seigo Yamashita, Michihiro Yoshimura, Jun-ichi Nitta

**Affiliations:** ∗ Division of Cardiology, Department of Internal Medicine, The Jikei University School of Medicine, Tokyo, Japan; † Department of Cardiology, Japanese Red Cross Saitama Hospital, Saitama, Japan; ‡ Department of Social Medicine, The Jikei University School of Medicine, Tokyo, Japan; § Department of Cardiology, Sakakibara Memorial Hospital, Tokyo, Japan

**Keywords:** Atrial fibrillation, Catheter ablation, Complication, Diarrhea, Hypokalemia

## Introduction

Percutaneous catheter ablation is an established intervention for the treatment of atrial fibrillation (AF). In line with the widespread use of this technique, its complications have also received attention.^1^^,^^2^ Although rare, cardiac tamponade, cerebral embolism, pulmonary vein stenosis, esophageal fistulas, and other serious complications may occur. Gastrointestinal (GI) symptoms related to the upper GI tract, such as esophageal damage/fistula and gastric hypomotility, are well known,^3^^,^^4^ while lower digestive tract symptoms are less known and underestimated. We herein report 3 cases of persistent diarrhea that occurred after catheter ablation for AF.

## Case reports

### Case 1

**Figure 1:**
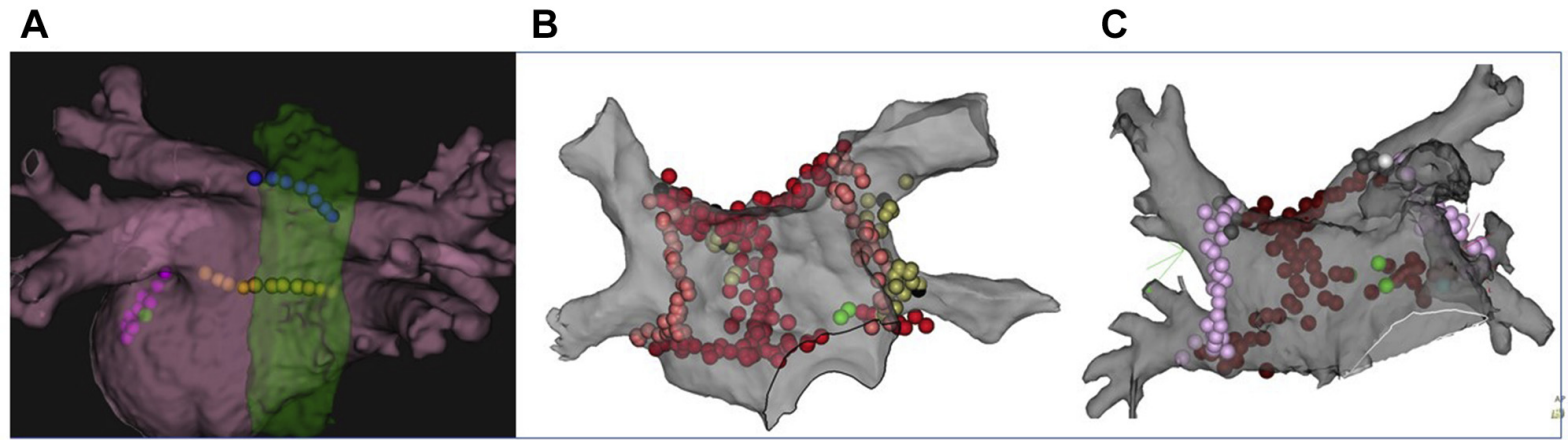
Ablation points on a 3-D mapping view in each case. **A:** Case 1: The patient underwent linear ablation on the roof (*blue*) and bottom (*yellow*) of the left atrial posterior wall to establish box isolation in the third procedure; both points traversed the anterior wall of the esophagus (*shown in green*). Another linear ablation at the mitral isthmus was performed (*pink*). Pulmonary vein (PV) isolation had been established in the previous procedures. **B, C:** Cases 2 and 3: In their first procedure, circumferential linear ablation was performed to isolate the ipsilateral PVs in 2-by-2 fashion and at the roof and bottom of the left atrial posterior wall to establish the box isolation.

After giving his written informed consent, a 43-year-old man underwent a third session of radiofrequency (RF) catheter ablation for drug-resistant, symptomatic paroxysmal AF at the Jikei University Hospital in April 2018. In the first and second ablation procedure in November 2016 and May 2017, respectively, all 4 pulmonary vein (PV) isolation, cavotricuspid isthmus ablation, superior vena cave isolation (in the first procedure), and additional linear ablations in the left atrium (LA) (roof line and mitral isthmus line, in the second procedure) were successfully performed. In the index (third) procedure for recurrent AF and atrial tachycardia, additional RF applications were performed to the reconducted roof line, LA bottom line (LA posterior box isolation was established) using a saline-irrigated catheter with a maximum RF energy of 35 watts (Figure 1). During the bottom-line ablation, the esophageal temperature transiently increased to 43.1°C. No complications were detected during the procedure, and the patient was discharged with oral anticoagulation on the second day after the procedure without any GI symptoms. From the 10th day after the procedure, diarrhea occurred and worsened day by day. Antiflatulence drugs did not improve his diarrhea. By the 50th day, he had lost 12 kg of body weight (77 kg to 65 kg) and had a serum potassium value of 2.7 mmol/L. Despite the oral administration of a potassium replacement drug (KCl tablet, 600 mg/day), he became unable to walk owing to lower extremity weakness and was transported to the emergency room. His serum potassium value at admission was 1.7 mmol/L (Table 1). His abnormal electrolyte values gradually normalized with intravenous replacement of potassium, inorganic phosphorus, and magnesium during the first week of admission, which improved his weakness and enabled him to walk. No abnormal findings suggesting the presence of inflammatory bowel disease were observed by colonoscopy. The stubborn diarrhea was relieved after the oral administration of ramosetron hydrochloride (2.5–5 μg/day). He was discharged on the 15th day with continuous oral potassium (tablet) and ramosetron hydrochloride, which was tapered during the subsequent 3 months. He has been completely free from palpitations and recurrent AF during 2 years of follow-up.

**Table 1:** Salient patient details

Patient no.	Age	Sex	HT/BW (BMI)	Type of AF	No. of sessions	ABL strategy	Max RF power (W) at LA posterior wall	Max esoph. temp. (°C)	LAD/EF on UCG (mm/%)	Occurrence of diarrhea after ABL	Type of diarrhea	Duration of diarrhea	Serum K (pre/min)	CRP during diarrhea
1	43	M	179/77.4 (24.2)	PAF	3rd	PVI BOX SVCI	35	43.1	34/60	10 d	Watery	5 mo	3.8/1.7	0.25
2	40	M	174/73.3 (24.2)	PAF	1st	PVI BOX	25	---	30/55	21 d	Watery	9 mo	4.1/3.7	0.1
3	44	M	170/70 (24.2)	Per-AF	1st	PVI BOX	25	---	31/60	14 d	Watery	12 mo	4.4/3.8	1.5

ABL = ablation; AF = atrial fibrillation; BOX = box isolation; CRP = C-reactive protein; EF = ejection fraction; K = potassium; LA = left atrium; LAD = left atrial dimension; Max esoph. temp. = maximum esophageal temperature; PAF = paroxysmal atrial fibrillation; Per-AF = persistent atrial fibrillation; pre/min = preprocedure and minimum value; PVI = pulmonary vein isolation; RF = radiofrequency; SVCI = superior vena cava isolation; --- = not measured; UCG = ultrasonic echocardiograph.

### Case 2

At Saitama Red Cross Hospital, a 40-year-old man with paroxysmal AF underwent PV isolation and a posterior wall isolation procedure using a standard irrigation catheter for the RF application (Figure 1, Table 1), with the maximum energy output of 25 watts (20 watts in front of the esophagus). A baseline examination (blood, X-ray photograph, echocardiography) revealed no abnormal findings. Although no complications were detected during the procedure or admission period, he suffered from severe watery diarrhea from the 21st day after the procedure. In the 9 months for which the diarrhea persisted, he lost almost 20 kg (73.3 kg to 54 kg). Antiflatulence drugs did not improve the diarrhea. Upper GI endoscopy revealed no abnormal findings. His minimum serum potassium value was 3.7 (on the 92nd day after ablation). His diarrhea gradually relieved after the oral use of Tsumura-Kampo Rikkunshito extract granules and polycarbophil calcium (3 g/day), which have been shown to be effective for the intractable diarrhea owing to the irritable bowel syndrome or ulcerative colitis. He became free from AF and diarrhea during the 5 years of follow-up without any medications.

### Case 3

At the same hospital, a 44-year-old man with persistent AF underwent PV isolation and a posterior wall isolation procedure using a standard irrigation catheter for the RF application, with a maximum energy output of 25 watts (20 watts in front of the esophagus) (Figure 1, Table 1). His baseline examination (blood, X-ray photograph, echocardiography) revealed no abnormal findings. He was discharged on the second day after ablation without any complications. He suffered from severe watery diarrhea from the 14th day after the procedure and lost 10 kg (70 kg to 60 kg) during the subsequent 12 months with persistent diarrhea. Upper GI endoscopy revealed no abnormal findings. His minimum serum potassium value was 3.8 mmol/L (on the 210th day after ablation). His diarrhea was gradually relieved with the oral use of Tsumura-Kampo Rikkunshito extract granules. He became free from AF and diarrhea after 2 years of follow-up without any medications.

The patient in case 1 is the only patient among 4000 AF ablations (1/4000) in Jikei University Hospital and cases 2 and 3 are the only 2 cases among 5000 AF ablation cases (2/5000) in the Japanese Red Cross Saitama Hospital in which the intractable diarrhea appeared following the catheter ablation of AF. Therefore, the estimated frequency of this complication will be 0.025% to 0.04%. None of the 3 cases demonstrated the bradycardia/hypotension or vagal responses during the box isolation procedure.

## Discussion

This report describes a lesser-known adverse effect—persistent diarrhea—of catheter ablation of AF. This probably results from the effects of RF energy delivered to the left atrium on the periesophageal vagal plexus. Although upper GI complications after catheter ablation of AF, such as esophageal damage/fistula, and gastric hypomotility, are well known,^3^^,^^4^ less is known about lower digestive tract symptoms, including diarrhea, and its incidence may be underestimated.

The clinical presentations of all 3 patients were very similar and the common features of these 3 cases were as follows: (1) all were relatively young men with a good physique, (2) the patients underwent box isolation by RF ablation using an irrigation catheter, (3) diarrhea appeared 2–3 weeks (10–21 days) after the ablation procedure, (4) persistent severe (watery) diarrhea lasted 3 months to 1 year, (5) all patients showed a large amount of weight loss (5–15 kg), (6) hypokalemia was present, (7) there was an absence of marked elevation of the inflammatory response (C-reactive protein), and (8) diarrhea gradually responded to ramosetron or Tsumura Kampo Rikkunshito extract granules and polycarbophil calcium.

Although there have been various reports on the complications of AF ablation, diarrhea is generally not included in the list of complications.^1^^,^^2^ As for GI complications, Bunch and colleagues^5^ reported the cases of 3 patients with vagus nerve injury after posterior atrial RF ablation, 2 of whom complained of upper GI symptoms: abdominal bloating, distention, early satiety, appetite loss, and green-colored stool. In a recent report, Jacobs and colleagues^6^ described various types of vagus nerve injury syndromes after catheter ablation for AF. Moderate diarrhea was observed in 24% of patients at baseline, and 31% and 20% of patients at 1 and 3 months after ablation, respectively. As for the severe diarrhea, the prevalence at baseline (6%) decreased to 5% and 2% at 1 and 3 months after the procedure, respectively. In previous articles, diarrhea has not been regarded as a serious complication after ablation procedures.

Different from the catheter ablation of arrhythmias, diarrhea is a well-known complication after laparoscopic antireflux surgery or paraesophageal hernia repair.^7^, ^8^, ^9^ The pathophysiology of postvagotomy diarrhea has been shown to be a complex process: The rapid influx of a hyperosmolar load into the small bowel owing to gastric incontinence and accelerated small bowel transit play major roles, and other contributing factors include bacterial overgrowth as a consequence of reduced gastric acidity and impaired resorption of bile acids as a result of rapid small-bowel transit.^10^, ^11^, ^12^ The management of diarrhea after vagotomy will be better on its mechanism. In addition to the basic treatment of withholding fluids and the use of hydrophilic colloids and antidiarrheal agents, bile acid–binding resin cholestyramine and broad-spectrum antibiotics to reduce bacterial overgrowth may be needed in some patients.^13^, ^14^ Surgical procedures should be reserved for patients in whom medical treatment fails.^15^

The progression of hypokalemia is one of the striking features of our patients, especially in the first case. The patient developed severe hypokalemia with quadriplegia as a consequence of persistent watery diarrhea and lost 15 kg of body weight, despite receiving potassium replacement therapy. We should realize that diarrhea and abnormal bowel movement should not be overlooked and should be treated carefully as a possibly fatal complication of catheter ablation for AF.

All 3 cases in this paper underwent box isolation with the index procedure, which isolates both pulmonary veins and the left atrial posterior wall (Figure 1). Both the roof and bottom lines traverse the anterior wall of the esophagus and can be harmful to the esophagus itself as well as the surrounding vagal nerves. In the 3 cases in this report, the esophageal temperature was monitored during the RF energy application in the first case, but not in the other 2 cases. Since there is still controversy regarding the usefulness of esophageal temperature monitoring during RF application, we should not have too much confidence in the value of esophageal temperature in avoiding esophagus-related complications.

It is noteworthy that diarrhea appeared after a substantial period (10–21 days after the procedure) in all cases. Both the patient and the attending physician may be unable to realize that diarrhea is a complication of the ablation procedure and the patient is likely to undergo gastroenterological examinations to investigate possible hidden diseases, such as inflammatory bowel disease. This is the first report demonstrating that persistent diarrhea is a possible complication of left atrial catheter ablation. Awareness and recognition of this complication may result in a better understanding of the magnitude and clinical spectrum of this complication.

**Key Teaching Points**

• Although upper gastrointestinal complications after catheter ablation of atrial fibrillation (AF), such as esophageal damage/fistula and gastric hypomotility, are well known, less is known about lower digestive tract symptoms, including diarrhea.
• The common clinical presentations of cases with intractable diarrhea are as follows: relatively young patients with a good physique, box isolation by radiofrequency ablation using an irrigation catheter, diarrhea appeared 2–3 weeks after the ablation procedure and lasted 3 months to 1 year, large amount of weight loss (5–15 kg), and hypokalemia (minimum value of 1.7 mmol/L).
• Diarrhea and abnormal bowel movement should not be overlooked and should be treated carefully as a possibly fatal complication of catheter ablation for AF.

## References

[1] Muthalaly R.G., John R.M., Schaeffer B. (2018). Temporal trends in safety and complication rates of catheter ablation for atrial fibrillation. J Cardiovasc Electrophysiol.

[2] Murakawa Y., Yamane T., Goya M. (2018). Japanese Heart Rhythm Society Members. Influence of substrate modification in catheter ablation of atrial fibrillation on the incidence of acute complications: analysis of 10 795 procedures in J-CARAF Study 2011-2016. J Arrhythm.

[3] Kapur S., Barbhaiya C., Deneke T., Michaud G.F. (2017). Esophageal injury and atrioesophageal fistula caused by ablation for atrial fibrillation. Circulation.

[4] Lakkireddy D., Reddy Y.M., Atkins D. (2015). Effect of atrial fibrillation ablation on gastric motility: The Atrial Fibrillation Gut Study. Circ Arrhythm Electrophysiol.

[5] Bunch T.J., Ellenbogen K.A., Packer D.L., Asirvatham S.J. (2008). Vagus nerve injury after posterior atrial radiofrequency ablation. Heart Rhythm.

[6] Jacobs V., May H.T., Crandall B.G. (2018). Vagus nerve injury symptoms after catheter ablation for atrial fibrillation. Pacing Clin Electrophysiol.

[7] Hashimi S., Bremner R.M. (2015). Complications following surgery for gastroesophageal reflux disease and achalasia. Thorac Surg Clin.

[8] Ukleja A., Woodward T.A., Achem S. (2002). Vagus nerve injury with severe diarrhea after laparoscopic antireflux surgery. Dig Dis Sci.

[9] Trus T.I., Bax T., Richardson W.S. (1997). Complications of laparoscopic paraesophageal hernia repair. J Gastrointest Surg.

[10] McKelvey S.T. (1970). Gastric incontinence and post-vagotomy diarrhoea. Br J Surg.

[11] Ladas S.D., Isaacs P.E., Quereshi Y., Sladen G. (1983). Role of the small intestine in postvagotomy diarrhea. Gastroenterology.

[12] Blake G., Kennedy T.L., McKelvey S.T. (1983). Bile acids and post-vagotomy diarrhoea. Br J Surg.

[13] Condon J.R., Robinson V., Suleman M.I., Fan V.S., McKeown M.D. (1975). The cause and treatment of postvagotomy diarrhoea. Br J Surg.

[14] Allan J.G., Russel R.I. (1977). Cholestyramine in treatment of postvagotomy diarrhoea: Double-blind controlled trial. BMJ.

[15] Cuschieri A. (1986). Surgical management of severe intractable postvagotomy diarrhoea. Br J Surg.

